# Adverse drug reaction and causality assessment scales

**DOI:** 10.4103/0970-2113.80343

**Published:** 2011

**Authors:** Syed Ahmed Zaki

**Affiliations:** *Department of Pediatrics, Lokmanya Tilak Municipal General Hospital and Medical College, Sion, Mumbai - 400 022, India. E-mail: drzakisyed@gmail.com*

Sir,

I read with interest the articles by Gupta *et al* and Gulati *et al* on adverse drug reactions of antituberculous drugs.[1,2] I would like to make the following comments.

Adverse drug reactions (ADRs) are a major cause of morbidity, hospital admission, and even death. Hence it is essential to recognise ADRs and to establish a causal relationship between the drug and the adverse event. It is desirable that ADRs should be objectively assessed and presented based on an acceptable “Probability Scale.” Many causality methods have been proposed to assess the relationship between a drug and an adverse event in a given patient, ranging from short questionnaires to comprehensive algorithms. The idea of creating a standardized assessment for the relationship-likelihood of case reports of suspected ADRs was in the hope that this would, in a structured way, lead to a reliable reproducible measurement of causality. The causality assessment system proposed by the World Health Organization Collaborating Centre for International Drug Monitoring, the Uppsala Monitoring Centre (WHO–UMC), and the Naranjo Probability Scale are the generally accepted and most widely used methods for causality assessment in clinical practice as they offer a simple methodology.[3,4] The above scales are structured, transparent, consistent, and easy to apply assessment methods. Table 1 summarizes the “Naranjo ADR Probability Scale,” which has gained popularity among clinicians because of its simplicity.[3] The WHO–UMC causality system takes into account the clinical-pharmacologic aspects, whereas previous knowledge of the ADR plays a less prominent role. Table 2 summarizes the WHO–UMC Probability Scale.[4]

**Table 1:** Naranjo ADR probability scale—items and score

Question	Yes	No	Don’t know
Are there previous conclusion reports on this reaction?	+1	0	0
Did the adverse event appear after the suspect drug was administered?	+2	–1	0
Did the AR improve when the drug was discontinued or a specific antagonist was administered?	+1	0	0
Did the AR reappear when drug was re-administered?	+2	–1	0
Are there alternate causes [other than the drug] that could solely have caused the reaction?	–1	+2	0
Did the reaction reappear when a placebo was given?	–1	+1	0
Was the drug detected in the blood [or other fluids] in a concentration known to be toxic?	+1	0	0
Was the reaction more severe when the dose was increased or less severe when the dose was decreased?	+1	0	0
Did the patient have a similar reaction to the same or similar drugs in any previous exposure?	+1	0	0
Was the adverse event confirmed by objective evidence?	+1	0	0

Scoring for Naranjo algorithm: >9 = definite ADR; 5–8 = probable ADR; 1–4 = possible ADR; 0 = doubtful ADR.

**Table 2:** WHO–UMC causality categories

Causality term	Assessment criteria (all points should be reasonably complied)
Certain	Event or laboratory test abnormality, with plausible time relationship to drug intake Cannot be explained by disease or other drugs Response to withdrawal plausible (pharmacologically, pathologically) Event definitive pharmacologically or phenomenologically (ie, an objective and specific medical disorder or a recognized pharmacologic phenomenon) Rechallenge satisfactory, if necessary
Probable/likely	Event or laboratory test abnormality, with reasonable time relationship to drug intake Unlikely to be attributed to disease or other drugs Response to withdrawal clinically reasonable Rechallenge not required
Possible	Event or laboratory test abnormality, with reasonable time relationship to drug intake Could also be explained by disease or other drugs Information on drug withdrawal may be lacking or unclear
Unlikely	Event or laboratory test abnormality, with a time to drug intake that makes a relationship improbable (but not impossible) Disease or other drugs provide plausible explanation
Conditional/unclassified	Event or laboratory test abnormality More data for proper assessment needed, or Additional data under examination
Unassessable/unclassifiable	Report suggesting an adverse reaction Cannot be judged because information is insufficient or contradictory Data cannot be supplemented or verified

I humbly request the Editors that *Lung India* should use either of the above two scales while reviewing articles related to ADRs.
